# Identification of potentially common loci between childhood obesity and coronary artery disease using pleiotropic approaches

**DOI:** 10.1038/s41598-022-24009-8

**Published:** 2022-11-14

**Authors:** Lianke Wang, Qiang Zhang, Fei Xu, Anna Brickell, Qianyu Zhou, Bin Yang, Changqing Sun

**Affiliations:** 1 Department of Epidemiology and Biostatistics, College of Public Health, Zhengzhou University, Zhengzhou, 450001 Henan China; 2 College of Nursing and Health, Zhengzhou University, Zhengzhou, 450001 Henan China; 3 College of Medicine, University of Arkansas for Medical Sciences, Little Rock, AR 72205 USA

**Keywords:** Data integration, Genome informatics, Computational biology and bioinformatics, Genetics, Cardiology

## Abstract

Childhood obesity remains one of the most important issues in global health, which is implicated in many chronic diseases. Converging evidence suggests that a higher body mass index during childhood (CBMI) is significantly associated with increased coronary artery disease (CAD) susceptibility in adulthood, which may partly arise from the shared genetic determination. Despite genome-wide association studies (GWASs) have successfully identified some loci associated with CBMI and CAD individually, the genetic overlap and common biological mechanism between them remains largely unexplored. Here, relying on the results from the two large-scale GWASs (*n* = 35,668 for CBMI and *n* = 547,261 for CAD), linkage disequilibrium score regression (LDSC) was used to estimate the genetic correlation of CBMI and CAD in the first step. Then, we applied different pleiotropy-informed methods including conditional false discovery rate ($\mathrm{cFDR}$) and genetic analysis incorporating pleiotropy and annotation (GPA) to detect potentially common loci for childhood obesity and CAD. By integrating the genetic information from the existing GWASs summary statistics, we found a significant positive genetic correlation (${r}_{g}$ = 0.127, *p* = 2E−4) and strong pleiotropic enrichment between CBMI and CAD (*LRT* = 79.352, *p* = 5.2E−19). Importantly, 28 loci were simultaneously discovered to be associated with CBMI, and 13 of them were identified as potentially pleiotropic loci by $\mathrm{cFDR}$ and GPA. Those corresponding pleiotropic genes were enriched in trait-associated gene ontology (GO) terms “amino sugar catabolic process”, “regulation of fat cell differentiation” and “synaptic transmission”. Overall, the findings of the pleiotropic loci will help to further elucidate the common molecular mechanisms underlying the association of childhood obesity and CAD, and provide a theoretical direction for early disease prevention and potential therapeutic targets.

## Introduction

Childhood obesity has emerged as an important public health problem and threatens future health and longevity, including an increased risk of many chronic disorders and premature death^1–3^. In recent decades, the worldwide prevalence of childhood obesity is on the increase remarkably^2^, and more than 22% of children are overweight or obese in developed countries^3^. The data from the World Health Organization (WHO) shows that there are over 340 million overweight and 124 million obese children and adolescents in 2016^4^. BMI is an established and most commonly used index to quantitatively measure obesity and health. Numerous studies have demonstrated that a higher body mass index during childhood (CBMI) is associated with an increased risk of coronary artery disease (CAD) in later adult life^5–7^. Several lines of evidence suggested that this association may partially arise from the common genetic foundation between the two phenotypes^8,9^. However, the biological mechanisms underlying this relationship are still unclear.

Genome-wide association studies (GWASs) have been widely used to identify single nucleotide polymorphisms (SNPs) contributing to the variation of complex traits or diseases, including CBMI and CAD. Heritability studies indicated a strong genetic contribution to both CBMI (*h*^2^: 67–93%)^10^ and CAD (*h*^2^: 40–60%)^11^. To date, GWASs have identified 161 loci for CAD and 25 loci for CBMI, accounting for 15.1% and 3.6% of the phenotypic variance of CAD and CBMI, respectively^8,12^. This indicated the heritability explained by the variants previously reported was limited (especially for CBMI) and most of the heritability was probably not missing but undetected. Additionally, hundreds of SNPs associated with adult BMI or obesity-related traits had been identified, by contrast, less was well known about the genetic background of CBMI^13^. Meanwhile, evidence from previous researches suggested that common genetic variant existed in CBMI and CAD^9^, indicating shared genetic determinants or risk factors between the two phenotypes.

GWAS can uncover more genetic loci to further explain the heritability of complex phenotypes by increasing the sample size^14,15^, but it is commonly not feasible since recruiting additional participants and SNP genotyping is too costly. Moreover, GWASs are usually performed on a single trait or disease, rather than analyzing them concurrently. Therefore, pleiotropic methods without extensive additional subject recruitment requirements are effective to discover novel genetic variants associated with multiple diseases or traits. Pleiotropy is the phenomenon of a single locus influencing more than two distinct diseases or traits^16^. Now, with the active pleiotropic method development in this field, it is possible to comprehensively elucidate the genetic overlap and biological underpinnings between different traits or diseases^17^.

Considering the close relationship, high heritability, and potential pleiotropy between CBMI and CAD, we assume the two phenotypes are ideal for further analyses to improve the detection of associated loci and explore the common etiology. Additionally, the pleiotropy between adult BMI and CAD has been studied by our team before^18^. Owing to the genetic correlation of CBMI and adult BMI and the significance of early disease prevention, the shared genetic signals between CBMI and CAD are worthy of further study with larger sample size. In the present study, with summary statistics from the two large-scale existing GWAS datasets, we first utilized the linkage disequilibrium score regression (LDSC) method to estimate the overall genetic correlation between CBMI and CAD. Next, we applied the conditional false discovery rate ($\mathrm{cFDR}$) and the genetic analysis incorporating pleiotropy and annotation (GPA) approaches to explore whether CBMI shares susceptibility loci with CAD^19,20^ and discover some novel etiologic relationship between them. The current study will be a foundation of further researches about the two phenotypes and helpful for screening the shared genetic factor. Additionally, the potentially shared genetic influences and biological mechanisms can provide novel helpful strategies for preventing and treating CAD early.

## Results

### Genetic correlation

The LDSC results showed that the genome-wide SNP-based heritability was estimated to be 24.21% (*se* = 2.21%) for CBMI and 5.83% (*se* = 0.33%) for CAD. Additionally, the LDSC intercept was 0.9971 (*se* = 0.007) for CBMI and 0.9951 (*se* = 0.011) for CAD, demonstrating that the results of our analysis were reliable and not affected by the confounding factors from population stratification or relatedness. Next, using cross-trait LDSC with 1,053,840 common SNPs from filtered summary statistics, we found that there existed a significant positive genetic correlation between CBMI and CAD (${r}_{g}$ = 0.127, *p* = 2E−4), indicating the genetic overlap between the two phenotypes. Therefore, further analyses were implemented to explore the specific genetic mechanisms shared by CBMI and CAD.

### Pleiotropic enrichment

**Figure 1:**
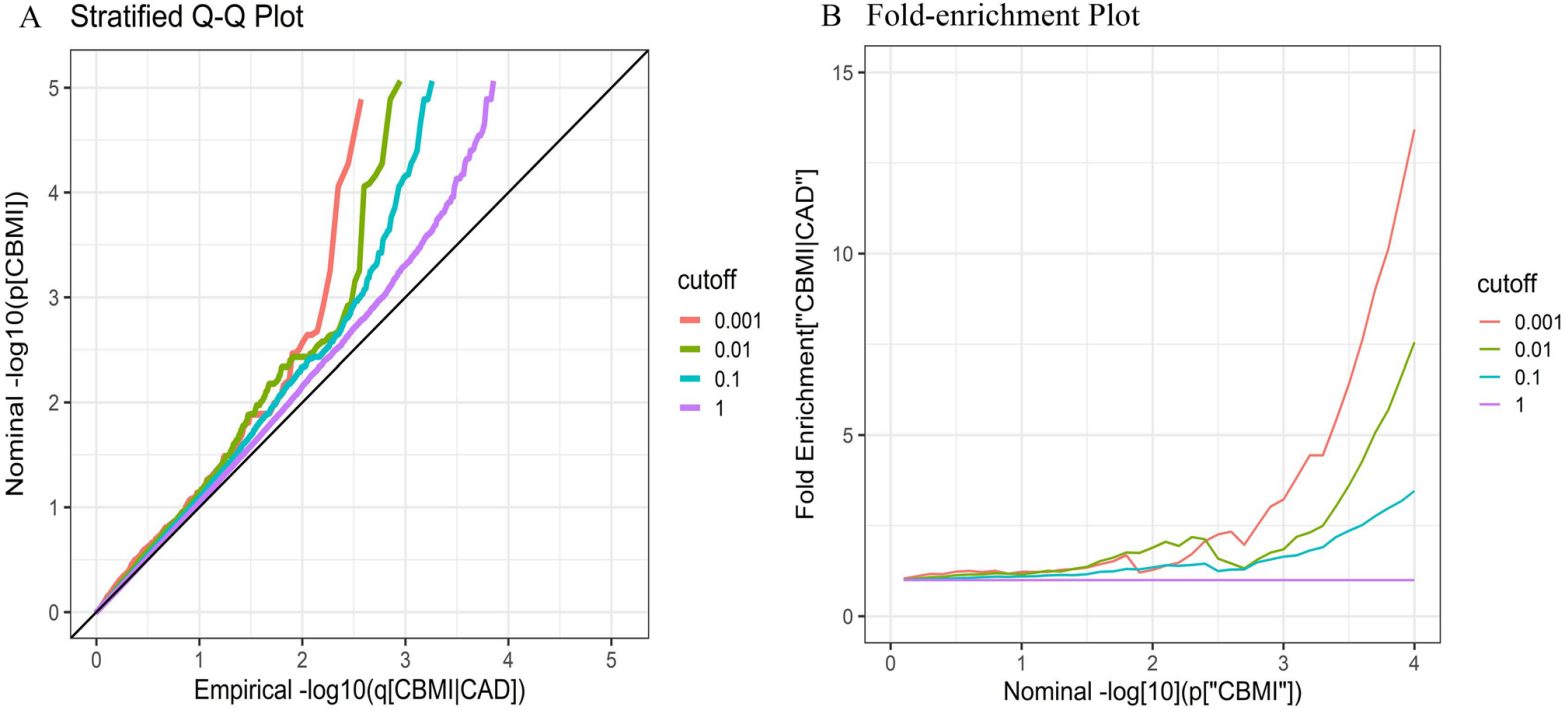
Stratified Q–Q plot (**A**) and Fold-enrichment plot (**B**). Stratified Q–Q plot: Nominal versus empirical ${-\mathrm{log}}_{10}(\mathrm{p})$ values in CBMI as a function of significance of association with CAD at different levels. Fold-enrichment plot: Enrichment versus nominal ${-\mathrm{log}}_{10}(\mathrm{p})$ values for CBMI as a function of significance of the association with CAD.

A stratified Q-Q plot for CBMI conditioned on different strengths of the association of CAD was shown in Fig. 1A. Strong pleiotropic enrichment between CBMI and CAD was revealed in the conditional Q-Q plot, as reflected by a large degree of leftward deflection from the null line under the successively decreasing thresholds. Earlier departures from the null line with higher levels of association with CAD demonstrate a greater proportion of true associations for a given nominal p-value. The fold-enrichment plot also displayed the genetic enrichment for CBMI conditioned on varying significance levels of CAD by observing the extent of upward shift from the reference line (Fig. 1B). Meanwhile, according to the LRT result (Table 1), we found that there was significantly pleiotropic enrichment between CBMI and CAD (the LRT *p*-value was 5.2E−19), which entirely supported the evidence provided from the above two different plots.

**Table 1:** Pleiotropy assessed between CBMI and CAD by GPA method.

	${\uppi }_{00}$	${\uppi }_{10}$	${\uppi }_{01}$	${\uppi }_{11}$	LRT	*p*-value
CBMI-CAD	0.857 (0.006)	0.048 (0.006)	0.090 (0.003)	0.005 (0.002)	79.352	5.2E−19

The values in the brackets are standard errors of the estimates. The last two columns provide the LRT statistics and p-values of hypothesis testing. The ${\uppi }_{00}$ represents the proportion of the SNPs not associated with CBMI or CAD; ${\uppi }_{10}$ and ${\uppi }_{01}$ represent the proportion of the SNP associated with CBMI and CAD, respectively; ${\uppi }_{11}$ represents the proportion of the SNP associated with CBMI and CAD.

### CBMI genetic loci identified with two methods

We identified 31 and 29 significant SNPs associated with CBMI according to the thresholds of $\mathrm{cFDR}$ < 0.05 and $\mathrm{fdr}.\mathrm{GPA}$ < 0.2, respectively. As illustrated in the conditional Manhattan plot for CBMI (Fig. S1), a total of 28 significant SNPs was identified to be associated with CBMI conditioned on CAD, which was annotated to 41 genes and located on 14 different chromosomes (Table S1). Among the 28 SNPs, 9 SNPs had *p*-values smaller than the genome-wide significance level (5E−8) and were considered to be associated with CBMI^21^, and 2 SNPs rs7127507 and rs7531118 were in the same LD block with the previously identified CBMI-associated SNPs rs17309874 (${r}^{2}$ = 0.6306) and SNP rs3101336 (${r}^{2}$ = 0.6805), respectively. These genetic variants were regarded as a replication of the previous CBMI-associated GWASs. The rest of 17 independent SNPs with p-values larger than the genome-wide significance threshold were considered novel SNPs to be associated with CBMI. For the 41 genes annotated by these SNPs, 29 genes were newly discovered in comparison with the original CBMI GWAS and previous CBMI-related studies. Other information on these 28 SNPs was presented in Table S1. Among the 41 genes detected with CBMI, most of them were enriched in CBMI-related GO terms such as “positive regulation of cellular process”, “limb morphogenesis” and “positive regulation of cell proliferation”. More detailed information on enrichment analysis was shown in Table 3. As for PPI results (Fig. S2), 28 genes including 21 novel ones were annotated in this protein network. The proteins encoded by these genes indirectly interacted with many other proteins encoded by CBMI-associated genes, proteins including *FTO*, *SEC16B*, *GNPDA2*, and *TFAP2B* had very close contact, which was proven to have a confirmed correlation with CBMI in original GWAS.

### Pleiotropic loci identified with two methods

**Figure 2:**
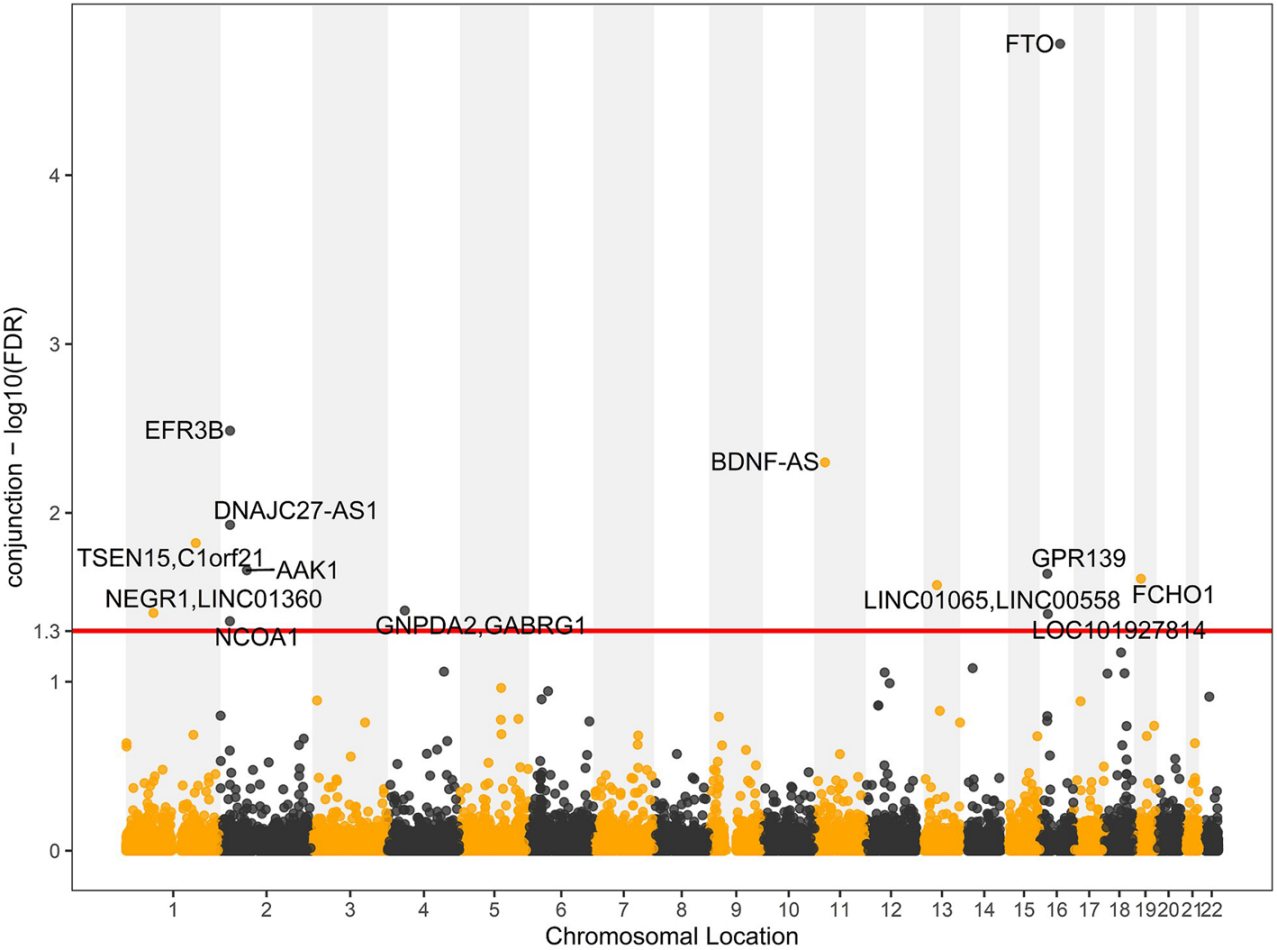
Conjunction Manhattan plot of ${-\mathrm{log}}_{10}(\mathrm{ccFDR})$ values for CBMI and CAD. The red line marks the ${-\mathrm{log}}_{10}(\mathrm{ccFDR})$ value of 1.3 corresponds to $\mathrm{ccFDR}$ of 0.05. The figure shows the genomic locations of shared variants and the overlapped genes between CBMI and CAD identified by $\mathrm{cFDR}$ and GPA method.

Using the two distinct methods with the thresholds of $\mathrm{ccFDR}$ < 0.05 and fdr11.GPA < 0.2, we identified 13 and 14 potentially pleiotropic SNPs, respectively. Taken together, 13 significant SNPs were detected to be associated with CBMI and CAD, these SNPs were annotated to 17 genes and located on 7 different chromosomes (Table 2 and Fig. 2). Most of the pleiotropic SNPs were situated in the intronic (54%) and intergenic (31%) regions while two were in the untranslated regions (15%). All of the 13 pleiotropic SNPs were not previously identified to be associated with both CBMI and CAD, only 3 SNP (rs9940128, rs11125884, and rs12429545) and 1 SNP rs13382133 passed genome-wide significance level in the original CBMI and CAD GWASs^12,21^, respectively. There were 8 SNPs (rs1866146, rs1996023, rs17736503, rs9940128, rs11125884, rs7127507, rs12429545 and rs7531118) previously reported to be associated with adult BMI-related traits^22,23^, and one SNPs rs9940128 was previously reported to be related with diabetes and high-density lipoprotein-cholesterol (HDL-C)^24,25^. All of the 17 genes these SNPs annotated to were novel because none of them were previously confirmed to be associated with both CBMI and CAD. More information on these SNPs and genes was presented in Table 2. A large number of the genes were enriched in CBMI and CAD-related terms such as “amino sugar catabolic process”, “regulation of brown fat cell differentiation” and “AP-2 adaptor complex binding”. More information on enrichment analysis was shown in Table 3. All the pleiotropic genes were imported into the STRING database, and only 9 genes were annotated in the PPI network. Those proteins encoded by the 9 genes were enriched into three disparate clusters (Fig. S3). For example, four proteins like *FTO*, *NEGR1*, *GABRG1*, and *GPR139* had the interplay in the *GNPDA2* cluster and the protein *FCHO1* directly interacted with the other protein *AAK1*.

**Table 2:** The pleiotropic loci identified by $\mathrm{cFDR}$ and GPA method ($\mathrm{ccFDR}$ < 0.05 and fdr11.GPA < 0.2).

Chr	SNP	Allel	Role	Gene	*P*.valueA	*P*.valueB	cFDR.AcB	cFDR.BcA	ccFDR	fdr11.GPA
chr16	rs9940128	G/A	Intronic	*FTO*	1.67E−05	9.25E−14	1.67E−05	2.45E−11	1.67E−05	0.001527
chr2	rs1866146	G/A	UTR3	*EFR3B*	0.000594	2.04E−06	0.003264	0.000802	0.003264	0.031703
chr11	rs7127507	T/C	ncRNA_intronic	*BDNF-AS*	0.000754	1.29E−05	0.005024	0.003705	0.005024	0.053765
chr2	rs11125884	A/G	ncRNA_intronic	*DNAJC27-AS1*	0.01177	2.73E−13	0.01177	4.88E−10	0.01177	0.109489
chr1	rs7536226	C/T	Intergenic	*TSEN15, C1orf21*	0.000247	5.31E−05	0.004194	0.015092	0.015092	0.064655
chr2	rs7420531	G/A	UTR3	*AAK1*	0.000533	8.77E−05	0.007644	0.021845	0.021845	0.088304
chr16	rs16969473	A/G	Intronic	*GPR139*	0.002157	8.22E−05	0.018119	0.022945	0.022945	0.126129
chr19	rs13382133	C/T	Intronic	*FCHO1*	7.67E−12	0.000558	1.1E−09	0.024548	0.024548	0.12562
chr13	rs12429545	G/A	Intergenic	*LINC01065, LINC00558*	0.02676	3.66E−11	0.02676	7.68E−08	0.02676	0.175974
chr4	rs1996023	T/G	Intergenic	*GNPDA2, GABRG1*	0.007574	2.73E−07	0.03787	0.00037	0.03787	0.094976
chr1	rs7531118	T/C	Intergenic	*NEGR1, LINC01360*	0.01305	1.29E−05	0.03915	0.007047	0.03915	0.182283
chr16	rs194546	G/A	ncRNA_intronic	*LOC101927814*	0.006605	8.62E−06	0.03963	0.007183	0.03963	0.123251
chr2	rs17736503	G/T	Intronic	*NCOA1*	0.007106	6.91E−05	0.04382	0.030049	0.04382	0.188942

The SNPs are listed with their chromosomal, allele, role, annotated genes, original *p*-value for each phenotype, cFDR value for each phenotype, ccFDR and fdr.11.GPA values. A means CAD and B means CBMI.

Chr: chromosom, ccFDR: conjunctional conditional false discovery rate, fdr.11GPA: false discovery rate of GPA when the SNP was associated with both phenotypes, GPA: genetic analysis incorporating pleiotropy and annotation.

**Table 3:** Gene functional annotation and enrichment result.

Go term	Pathway description	*P*-value	Combined score	Genes
CBMI GO:0048522	Positive regulation of cellular process	6.17E−04	41.68	*PKHD1, TFAP2B, AKAP6, CACUL1, TBX3, DYNAP*
GO:0035108	Limb morphogenesis	8.40E−04	329.02	*TFAP2B, TBX3*
GO:0008284	Positive regulation of cell proliferation	0.001672	36.78	*PKHD1, TFAP2B, CACUL1, TBX3, DYNAP*
GO:0072001	Renal system development	0.006516	83.23	*PKHD1, TFAP2B*
GO:1903779	Regulation of cardiac conduction	0.008333	69.71	*FPGT-TNNI3K, TNNI3K*
GO:0030136	Clathrin-coated vesicle	0.001126	99.35	*FCHO1, AAK1, RAB27B*
GO:0035612	AP-2 adaptor complex binding	8.55E−05	1305.44	*FCHO1, AAK1*
GO:0001104	RNA polymerase II transcription cofactor activity	6.79E−04	127.09	*NCOA1, TFAP2B, MED13L*
GO:0008528	G-protein coupled peptide receptor activity	0.007628	74.33	*PRLHR, GPR139*
CBMI and CAD GO:0046348	Amino sugar catabolic process	0.00509	1035.40	*GNPDA2*
GO:0090335	Regulation of brown fat cell differentiation	0.006781	734.36	*FTO*
GO:1901072	Glucosamine-containing compound catabolic process	0.008469	561.33	*GNPDA2*
GO:0006054	N-acetylneuraminate metabolic process	0.008469	561.33	*GNPDA2*
GO:0051932	Synaptic transmission, GABAergic	0.009313	500.15	*GABRG1*
GO:0030135	Coated vesicle	0.001633	212.68	*FCHO1, AAK1*
GO:0035612	AP-2 adaptor complex binding	1.42E−05	3750.98	*FCHO1, AAK1*
GO:0017162	Aryl hydrocarbon receptor binding	0.006781	734.36	*NCOA1*
GO:0008503	Benzodiazepine receptor activity	0.008469	561.33	*GABRG1*

## Discussion

In this study, by integrating two GWASs summary statistics of CBMI and CAD into $\mathrm{cFDR}$ and GPA framework, we identified 28 SNPs associated with CBMI, and 13 pleiotropic SNPs between CBMI and CAD. Among those annotated genes, 17 novel genes were found to be pleiotropic. In the present study, given the background and the focus of the study, we highlighted the SNPs identified with CBMI and the common SNPs associated with the two phenotypes.

The findings of some genetic signals in present study were consistent with the evidence from previous studies and the pleiotropic enrichment indicated a genetic relationship between CBMI and CAD. Moreover, most loci were not identified in previous genetic researches, indicating the effectivity of the two pleiotropic approaches on the detection of overlapped genetic variants. Meanwhile, the statistical power was largely improved, it was reported that there were about 15–20 times of power increased to detect more variants through a comparison of conditional vs. unconditional FDR approach^19^, and the statistical power of the GPA method was comparable to that of $\mathrm{cFDR}$ method^20^. More importantly, through contrastive analysis, we quantitatively investigated the pleiotropic enrichment evaluation between CBMI and CAD based on the LRT, and robust results were obtained. The findings of polygenic pleiotropy between CBMI and CAD enabled us to further illustrate the overlapped biological mechanisms underlying the different traits or diseases.

The shared genetic determination between CBMI and CAD emphasized the important role of CBMI in CAD development and supported the hypothesis that a common genetic basis existed between them. The two pleiotropy-informed methods jointly identified 13 shared SNPs annotated to 17 genes, these SNPs and corresponding genes were novel for the reason that no previous researches confirmed them to be related to both CBMI and CAD. These new findings could provide us a better understanding of the overlapped etiology between CBMI and CAD. Here, we will discuss some pleiotropic genetic signals for their potential biological function to further elucidate the phenotypic relevance.

The SNP rs9940128 (16q12.2) is the most statistically significant pleiotropic variant identified in our study, which is located in the intron region of the *FTO* gene, and was documented to be associated with CBMI in original GWAS^21^. Meanwhile, this variant also influencing adult BMI has been repeatedly confirmed in the earlier GWASs^23,26^. The *FTO* is a well-known gene involved in weight gain and obesity. Research based on the *FTO* mouse model further demonstrated that perturbing the *FTO* enzymatic activity could dysregulate genes related to energy metabolism, resulting in the imbalance of adipose tissue homeostasis in mice^27^. It provided clear evidence that *FTO* played important role in regulating food intake or energy metabolism. Furthermore, evidence from recent studies has emphasized the importance of *FTO* gene variation, revealing that they were associated with neuropsychiatric diseases^28^ besides metabolic disorders and human adiposity. Additionally, the *FTO* gene also played a potential role in cardiovascular diseases (CVD). A meta-analysis comprising 10 studies reported a significant association between the *FTO* gene and CVD risk, and this association was independent of BMI and other conventional CVD risk factors^29^. Subsequently, one study including a total of 970 samples that came from Pakistan demonstrated the variant in *FTO* gene was associated with CAD through affecting plasma glucose metabolism^30^. In addition, common variants in this gene were also associated with diabetes-related risk metabolic traits, such as raised fasting glucose, insulin and triglycerides, and lower HDL-C^31^. As described above, *FTO* gene was associated with CBMI and CAD may through these biological processes and molecular pathways, but further studies involving the exact functional characterization of this genetic signal are required to elucidate the precise mechanism behind their relationship.

The pleiotropic SNP rs1996023 (4p12) is located in the intergenic region between the *GNPDA2* gene and the *GABRG1* gene. The protein encoded by the *GNPDA2* gene belongs to an allosteric enzyme that catalyzes the deamination of glucosamine 6-phosphate and participates in the hexosamine signaling pathway^32,33^. SNPs in or close to the *GNPDA2* gene had been documented to be associated with adult BMI or childhood obesity^21^. One animal study also found that the *GNPDA2* gene played an important role in regulating body fat, weight, and energy metabolism^33^. Additionally, another study investigating the possible mechanism of the *GNPDA2* in adipogenesis discovered that overexpression of *GNPDA2* enhanced the accumulation of lipid droplets and knockout of the gene resulted in the reduction of accumulation of lipid droplets, reporting that *GNPDA2* may be a key gene in lipid and glucose metabolism^32^. Furthermore, through assessing the impact of the obesity-related loci on the known obesity complications found that the *GNPDA2* gene was potentially associated with diabetes^34^. It is widely known that the abnormal metabolism of lipid and glucose is a causal risk factor for cardiovascular diseases. A previous study also reported that the *GNPDA2* gene was highly expressed in the hypothalamus and brain, and played a vital role in the central nervous system processes of weight regulation^34^. The *GABRG1* gene is an integral membrane protein that encodes a protein belonging to the ligand-gated ionic channel family and participates in the inhibition of neural transmission^35^. *GABRG1* gene was reported to be implicated in neurological development conditions, including autism spectrum disorder^36^. The pathogenesis may involve the early development of the brain, affecting synaptic plasticity, neural transmission, and functional connectivity. There was evidence that children with neurodevelopmental disorders were at higher risk of developing depression^37^. Meanwhile, growing evidence shows depression is a risk factor for the development of CAD^38^. The *GNPDA2* and *GABRG1* genes increase the risk of susceptibility for CBMI and CAD may be through the same biological processes, but further experimentations are needed to determine the exact mechanism of the action of these genes.

The implementation of $\mathrm{cFDR}$ and GPA approaches on two large GWAS datasets let us successfully detect a large number of novel phenotype-associated genetic signals. Especially, an alternative pleiotropic method based on an independent algorithm was used simultaneously to ensure the reliability of the results. However, there are still a few limitations. First, the information about the precise effect size of the loci on the phenotype could not be provided, so it is impossible to determine which phenotype the locus has the greater influence on, but it can be inferred from the results of the original GWAS study. Second, the dataset of CAD is a meta-analysis of overwhelming European ancestry and a small fraction of other ancestries, we could not ensure uniformity of the ancestry due to the lack of data at the individual level, this may have an impact on the results. Nevertheless, the large sample size can contribute to the successful discovery of potential novel genetic variants. Third, independent replication is needed in this research. However, our CAD dataset is the largest to date which includes the dataset from the CARDIoGRAMplusC4D consortium and UKB. Moreover, we used the GWAS dataset of CAD from CARDIoGRAMplusC4D consortium for replication^39^, we observed a similar pleiotropic enrichment pattern and replicated 19 SNPs associate with CBMI and 3 pleiotropic SNPs associated with CBMI and CAD. These results illustrate that there is strong pleiotropic enrichment between CBMI and CAD and some common SNPs can be replicated across studies although with small sample size. Forth, both pleiotropic methods cannot identify the causal variants for the phenotypes of interest, but the question can be partially addressed in the future through the integration with other types of data^40^. Fifth, most of our findings are statistically significant and require further biological functional experiments and clinical replication studies to support our findings.

In summary, our results highlight the feasibility and improved power of the $\mathrm{cFDR}$ and GPA methods in detecting novel genetic pleiotropic loci between CBMI and CAD. The novel findings may facilitate the discovery of the shared genetic mechanisms underlying CBMI and CAD, and provide us novel insights into early disease prevention and treatment strategies.

## Materials and methods

### GWAS datasets

Our analyses were conducted on summary statistics from two independent GWASs for CBMI and CAD^12,21^. The CBMI GWAS dataset was derived from a GWAS meta-analysis of European descent involving 35,668 individuals performed by the EGG Consortium (http://www.egg-consortium.org/childhood-bmi.html). The CAD GWAS dataset was derived from a meta-analysis of 547,261 individuals (122,733 cases and 424,528 controls) of mainly European descent, which combined a GWAS dataset from the CARDIoGRAMplusC4D consortium with another GWAS dataset from the UK Biobank (UKB). The aggregate dataset of CAD was available through the CardiOmics website (https://www.cardiomics.net/download-data). The two datasets consist of summary statistics for each SNP, and their alleles, *p*-value, effect size, and direction of effect. More information such as study design, inclusion criteria of subjects, and quality control during the analysis is provided in the original study. There are no reduplicating subjects between the two datasets, avoiding the result of increased Type I error and biased effect estimates^41^.

We used summary-level statistics from the two GWA studies. All participants in the original study received informed consent, and the ethical approval had been obtained from their respective institutional review boards. The details can be found in the two GWASs^12,21^. This study was approved by the Ethical Committee of the Life Sciences of Zhengzhou University.

### Data processing

The two GWAS datasets were used here containing 2,499,691 SNPs for CBMI and 7,947,837 SNPs for CAD. Before conducting the following analysis, we extracted the SNPs and their corresponding variables (eg, alleles, effect sizes, *p*-values, sample sizes) from the two datasets for each phenotype. The HapMap3 CEU genotypes were used as the reference panel to label allele frequencies and calculate LD values between pairs of SNPs. For the LDSC analysis, the summary statistics were dealt with default SNP quality control (QC) filters (INFO > 0.9 and MAF > 0.01). After QC, a total of 1,054,231 and 1,181,375 SNPs were reserved for CBMI and CAD, respectively. Using cross-disease LDSC, 1,053,840 common SNPs were remained after merging. Before $\mathrm{cFDR}$ and GPA analysis, we first combined the common SNPs in the two datasets, then the SNPs were processed using the linkage disequilibrium (LD)-based pruning method by PLINK1.9. If the pairs of SNPs with LD values (${r}^{2}$) larger than 0.2, one of the SNPs with smaller minor allele frequency (MAF) was discarded^19^. Lastly, the remaining 128,749 common SNPs were prepared for the pleiotropic analysis. Genomic control (GC) corrected genomic inflation resulting from the potential population structure in the individual studies and the GWAS meta-analysis^42^. Therefore, there was no need for us to reuse the GC correction in this analysis.

### Statistical analyses

The Linux operating system and R 4.0.5 were used for data processing and statistical analysis. The LDSC, $\mathrm{cFDR}$, and GPA analyses were performed by “LDSC”, “$\mathrm{GWAScFDR}$” and “GPA” packages, respectively. The quantile–quantile (Q-Q) plot, fold-enrichment plot, and Manhattan plot were mainly generated by the “ggplot2” package.

### Estimation of genetic correlation

With the summary statistics from the two GWAS datasets, we first estimated the SNP heritability for each phenotype and evaluated the genetic correlation between CBMI and CAD using LDSC analysis. LDSC is a method of quantifying the contribution of polygenic heritability and confounding bias by examining the regression relationship between GWAS test statistics and LD to determine whether the inflation of test statistics is caused by polygenic effects or confounding bias^43,44^. The intercept of LDSC regression analysis can be used to determine whether there are confounding factors in the results. If the intercept is near 1, it means there is no confounding factor. For analysis of a single phenotype, LDSC can identify confounding factors and estimate heritability. For multiple phenotypes, the genetic correlation between phenotypes can be calculated according to the corresponding chi-square statistics^44^.

### Pleiotropic enrichment assessment

To assess the pleiotropic enrichment between the two phenotypes (CBMI and CAD), a stratified Q-Q plot was conducted by successively conditioning the principal phenotype on SNPs across varying levels of significance threshold for the given phenotype. The enrichment of the pleiotropic effect can be decided by the degree of deflection from the null line which is conditioned on the second phenotype. Meanwhile, we constructed a fold-enrichment plot to further confirm the pleiotropic enrichment between CBMI and CAD. The enrichment can be reflected by the larger degree of the upward shift from the null line when the p-values become smaller. Furthermore, in addition to the visual display of the pleiotropic enrichment above, we also used the likelihood ratio test (LRT) to quantitatively estimate the statistical significance of pleiotropic enrichment^20^.

### Calculation of $\mathbf{c}\mathbf{F}\mathbf{D}\mathbf{R}$, $\mathbf{c}\mathbf{c}\mathbf{F}\mathbf{D}\mathbf{R}$ and GPA

Both $\mathrm{cFDR}$ and GPA are well-established pleiotropic methods for incorporating GWAS data at the summary statistic level, which have been widely applied to many diseases or traits^45–48^. We briefly summarized both approaches in the following section. The $\mathrm{cFDR}$ is based on the Bayesian formula, identified as a posterior probability of an SNP being null for the principal phenotype given that the p-values for the two phenotypes (principal and conditional) are as small as or smaller than the observed ones. In the following formula, ${p}_{i}$ and ${p}_{j}$ are the observed p-values of a SNP for the principal and conditional phenotypes, respectively. ${H}_{0}^{\left(i\right)}$ means a null hypothesis that the SNP was not associated with the principal phenotype.

1 $$\mathrm{cFDR}\left({p}_{i}|{p}_{j}\right)=\mathrm{Pr}({H}_{0}^{\left(i\right)}|{P}_{i}\le {p}_{i} , {P}_{j}\le {p}_{j})$$

The $\mathrm{cFDR}$ threshold was set to 0.05, which meant the SNP was considered to be associated with the principal phenotype if the value is less than 0.05^19^. To identify an SNP to be pleiotropic, conjunction $\mathrm{cFDR}$ ($\mathrm{ccFDR}$) is defined based on $\mathrm{cFDR}$ and is calculated as the maximum value of the $\mathrm{cFDRs}$ for the two phenotypes^19^. Finally, the conditional and conjunction Manhattan plots were applied to present the location of the significant SNPs associated with CBMI, as well as pleiotropic SNP associated with CBMI and CAD.

The GPA method is another available statistical tool applied to detect the pleiotropic genetic effects between CBMI and CAD in the expectation of providing robust statistical evidence. This approach can use the GWAS summary results without annotation data, which has better comparability with the $\mathrm{cFDR}$ analysis results in our study. We took the intersection of the p-values of corresponding phenotypes as input, then a GPA model was fitted based on the provided p-value matrix data. The four-group model for two GWAS data sets was presented to implement the GPA analysis.

2 $$\begin{aligned} {\pi }_{00} & =\mathrm{Pr}\left({Z}_{j00}=1\right):\left({P}_{j1}|{Z}_{j00}=1\right)\sim U\left[\mathrm{0,1}\right],\left({P}_{j2}|{Z}_{j00}=1\right)\sim U\left[\mathrm{0,1}\right], \\ {\pi }_{10}& =\mathrm{Pr}\left({Z}_{j10}=1\right):\left({P}_{j1}|{Z}_{j10}=1\right)\sim Beta\left[{\alpha }_{1},1\right],\left({P}_{j2}|{Z}_{j10}=1\right)\sim U\left[\mathrm{0,1}\right], \\ {\pi }_{01} & =\mathrm{Pr}\left({Z}_{j01}=1\right):\left({P}_{j1}|{Z}_{j01}=1\right)\sim U\left[\mathrm{0,1}\right],\left({P}_{j2}|{Z}_{j01}=1\right)\sim Beta\left[{\alpha }_{2},1\right], \\ {\pi }_{11}&=\mathit{Pr}\left({Z}_{j11}=1\right):\left({P}_{j1}|{Z}_{j11}=1\right)\sim Beta\left[{\alpha }_{1},1\right],\left({P}_{j2}|{Z}_{j11}=1\right)\sim Beta\left[{\alpha }_{2},1\right] \end{aligned}$$

The latent variables ${Z}_{j}$ represent the association between the j-th SNP and the two phenotypes, ${Z}_{j00}=1$ means the SNP is associated with neither of them, ${Z}_{j10}=1$ and ${Z}_{j01}=1$ means the SNP is only associated with one of the two phenotypes, and ${Z}_{j11}=1$ means the SNP is associated with both. The $U\left[\mathrm{0,1}\right]$ and $Beta\left[{\alpha }_{k},1\right]$ means the p-values from the null group (Uniform distribution) and non-null group (Beta distribution, where 0 < ${\alpha }_{k}$  < 1, k = 1,2), respectively. The Expectation–Maximization (EM) algorithm was used to estimate the statistical inference of the model parameters and determine the SNP ranking^20^. Meanwhile, using the LRT-based hypothesis testing for pleiotropy, we calculated the value of $\mathrm{fdr}.\mathrm{GPA}$ (false discovery rate of GPA) to decide which variant to be associated with CBMI and the value of fdr11.GPA to determine the common variants between CBMI and CAD. We used the same the GPA significance threshold of 0.2 according to the criterion set in the original paper, which means this SNP is associated with the corresponding phenotype if the value is less than 0.2^20^.

### Definition of potentially novel SNP

The SNP was defined as potentially novel one should not have been reported in the previous GWASs and was not in linkage disequilibrium (LD) (${r}^{2}$ ≥ 0.6) with the previously GWASs confirmed SNPs. We mainly searched GWAS Catalog (https://www.ebi.ac.uk/gwas/) and PhenoScanner (http://www.phenoscanner.medschl.cam.ac.uk/) to determine the SNP and LD value (${r}^{2}$). If the value of ${r}^{2}$ was greater than 0.6, it means the two SNPs were in high LD and the SNP identified was considered to be reported or replicated.

### Functional analysis of the identified genes

To explore the functional role of the identified genes, we performed a comprehensive gene set enrichment analysis using Enrichr (http://amp.pharm.mssm.edu/Enrichr/)^49^. The significant genes were annotated and clustered in three main categories (biological processes, cellular components, and molecular functions) to clarify polygenic associations and determine whether the implicated genes were involved in a biological process. To further reveal and visualize the functional partnership and interaction of identified genes, protein–protein interaction (PPI) network was established by utilizing the STRING database (http://string-db.org/), which could construct the corresponding protein association networks by comprising known and predicted associations^50^. The above analyses enabled us to get a systematical evaluation of the underlying biology and relevance between the genes enriched in clusters, especially enhanced our understanding of the biological underpinnings of potential associations between different phenotypes.

## Supplementary Information

Supplementary Information.

## Data Availability

The data that support the findings of the current study are openly available in the Early Growth Genetics Consortium at http://www.egg-consortium.org/childhood-bmi.html and CardiOmics.net at https://www.cardiomics.net/download-data.
