# Supplementary material for: Identification of potentially common loci between childhood obesity and coronary artery disease using pleiotropic approaches
Source: Sci Rep. 2022 Nov 14;12:19513. doi: 10.1038/s41598-022-24009-8 (PMC9663585; doi:10.1038/s41598-022-24009-8)
Supplement: Supplementary file 1 — Supplementary Information. [file 41598_2022_24009_MOESM1_ESM.docx]

supplementary Material

# Supplementary Figures and Tables

**Fig. S1** Conditional Manhattan plot of ${-log}_{10} (cFDR)$ values for CBMI given CAD

**Fig.S2** Protein-protein interactions between CBMI-associated genes

**Fig. S3** Protein-protein interactions between pleiotropic genes

**Table S1** The CBMI-associated loci given CAD identified by $\mathrm{cFD}R$ and GPA method ($\mathrm{cFDR}<0.05$and $fdr.GPA$<0.2)


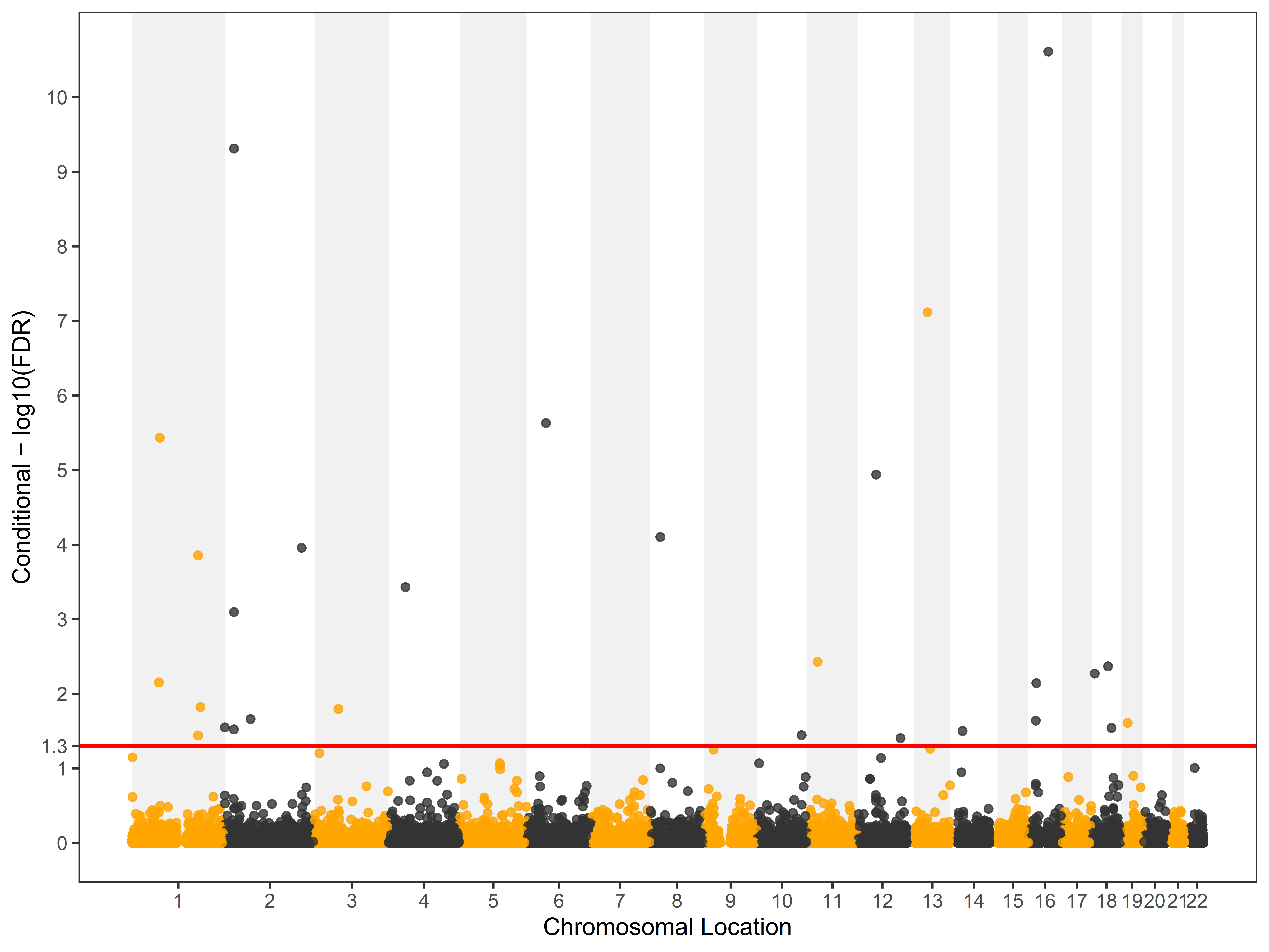


**Fig. S1** Conditional Manhattan plot of ${-log}_{10} (cFDR)$ values for CBMI given CAD.

The red line marks the ${-log}_{10} (cFDR)$ value of 1.3 corresponds to $\mathrm{cFDR}$ of 0.05. The figure shows the genomic locations of CBMI-associated SNPs identified by $\mathrm{cFDR}$ and GPA method.


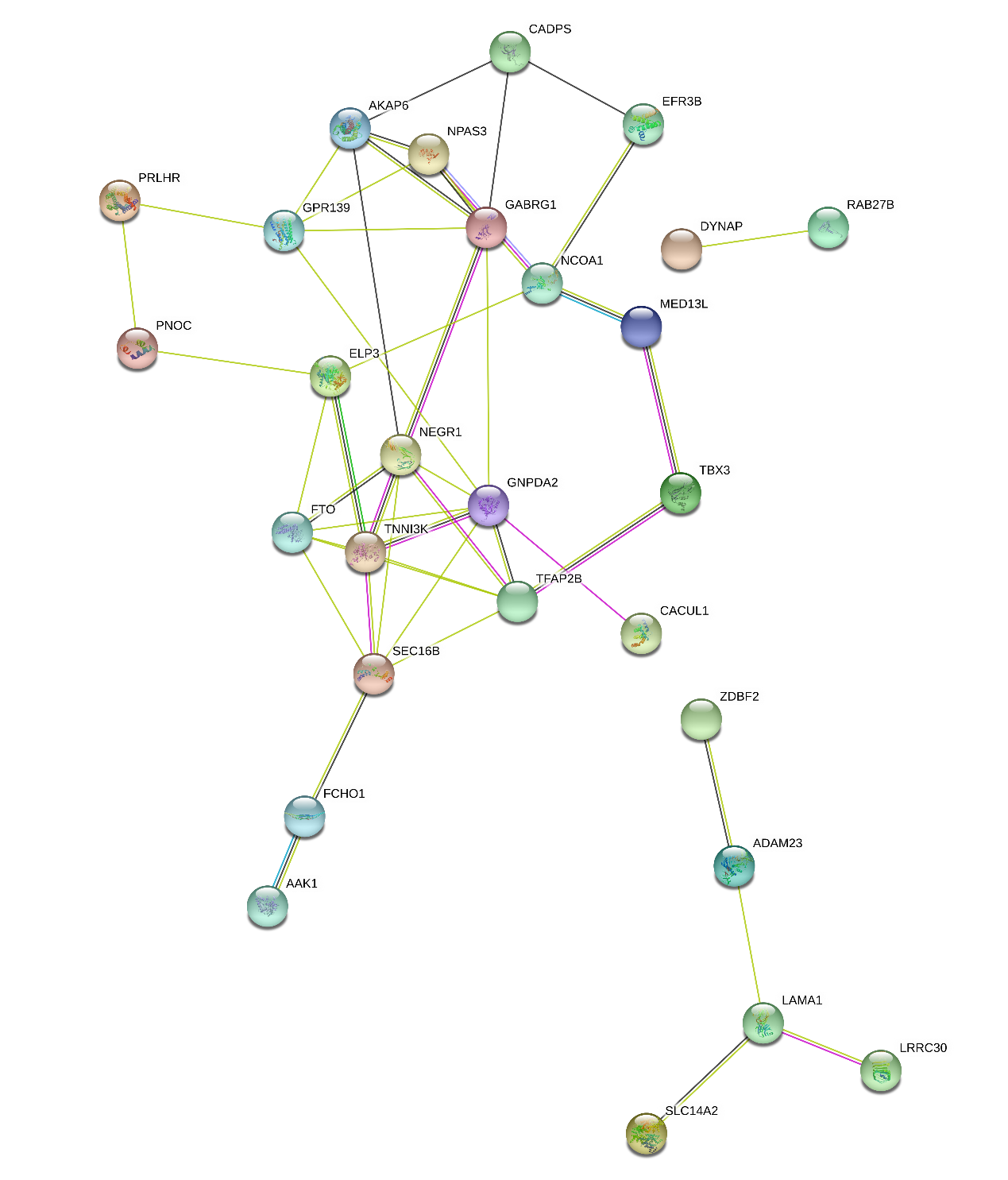


**Fig. S2** Protein-protein interactions between CBMI-associated genes.

Network nodes represented proteins produced by the identified genes and edges represented protein–protein associations.


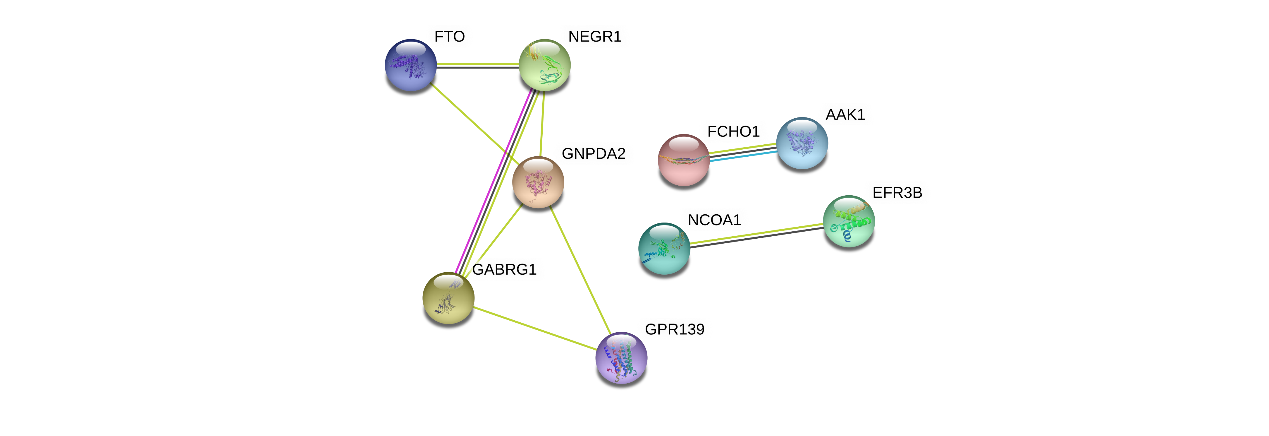


**Fig. S3** Protein-protein interactions between pleiotropic genes.

Network nodes represented proteins produced by the identified genes and edges represented protein–protein association.

**Table S1** The CBMI-associated loci given CAD identified by $\mathrm{cFD}R$ and GPA method ($\mathrm{cFDR}<0.05$and $fdr.GPA$<0.2)

| Chr | SNP | Allele | Role | Gene | SNP type | Gene type | *P*.valueB | cFDR.BcA | fdr.GPA |
| --- | --- | --- | --- | --- | --- | --- | --- | --- | --- |
| chr16 | rs9940128 | G/A | intronic | *FTO* | Confirmed | Confirmed | 9.25E-14 | 2.45E-11 | 8.25E-06 |
| chr2 | rs11125884 | A/G | ncRNA_intronic | *DNAJC27-AS1* | Confirmed | Confirmed | 2.73E-13 | 4.88E-10 | 3.57E-05 |
| chr13 | rs12429545 | G/A | intergenic | *LINC01065, LINC00558* | Confirmed | Novel, novel | 3.66E-11 | 7.68E-08 | 0.000417 |
| chr6 | rs2635727 | T/C | intergenic | *TFAP2B, PKHD1* | Confirmed | Confirmed, novel | 5.84E-10 | 2.34E-06 | 0.002263 |
| chr1 | rs7553348 | G/A | intronic | *FPGT-TNNI3K, TNNI3K* | Confirmed | Confirmed, confirmed | 1.97E-10 | 3.68E-06 | 0.002486 |
| chr12 | rs1031477 | C/T | intronic | *NCKAP5L* | Confirmed | Confirmed | 4.57E-09 | 1.15E-05 | 0.004377 |
| chr8 | rs13253111 | A/G | intergenic | *ELP3, PNOC* | Confirmed | Confirmed, novel | 4.13E-09 | 7.89E-05 | 0.010218 |
| chr2 | rs13387838 | G/A | intergenic | *ZDBF2, ADAM23* | Confirmed | Novel, novel | 2.40E-08 | 0.00011041 | 0.01428 |
| chr1 | rs2902210 | T/C | intronic | *SEC16B* | Confirmed | Confirmed | 3.72E-08 | 0.00013866 | 0.015805 |
| chr4 | rs1996023 | T/G | intergenic | *GNPDA2, GABRG1* | Novel | Confirmed, novel | 2.73E-07 | 0.00037019 | 0.012176 |
| chr2 | rs1866146 | G/A | UTR3 | *EFR3B* | Novel | Novel | 2.04E-06 | 0.00080188 | 0.015605 |
| chr11 | rs7127507 | T/C | ncRNA_intronic | *BDNF-AS* | LD (rs17309874) | Novel | 1.29E-05 | 0.00370517 | 0.035306 |
| chr18 | rs1552328 | T/G | intronic | *SLC14A2* | Novel | Novel | 3.49E-06 | 0.00427367 | 0.056552 |
| chr18 | rs8094220 | G/A | intergenic | *LAMA1, LRRC30* | Novel | Novel, novel | 4.09E-06 | 0.00535949 | 0.074385 |
| chr1 | rs7531118 | T/C | intergenic | *NEGR1, LINC01360* | LD (rs3101336) | Novel, novel | 1.29E-05 | 0.00704675 | 0.074583 |
| chr16 | rs194546 | G/A | ncRNA_intronic | *LOC101927814* | Novel | Novel | 8.62E-06 | 0.00718296 | 0.050065 |
| chr1 | rs7536226 | C/T | intergenic | *TSEN15, C1orf21* | Novel | Novel, novel | 5.31E-05 | 0.01509176 | 0.056017 |
| chr3 | rs17356252 | T/C | intronic | *CADPS* | Novel | Novel | 4.68E-06 | 0.01600835 | 0.142431 |
| chr2 | rs7420531 | G/A | UTR3 | *AAK1* | Novel | Novel | 8.77E-05 | 0.02184477 | 0.074195 |
| chr16 | rs16969473 | A/G | intronic | *GPR139* | Novel | Novel | 8.22E-05 | 0.02294466 | 0.091653 |
| chr19 | rs13382133 | C/T | intronic | *FCHO1* | Novel | Novel | 0.000558 | 0.0245476 | 0.12562 |
| chr2 | rs1320366 | C/G | intergenic | *LINC01874, LINC01875* | Novel | Novel, novel | 2.21E-05 | 0.02824933 | 0.17865 |
| chr18 | rs7237747 | C/G | intergenic | *DYNAP, RAB27B* | Novel | Novel, confirmed | 3.98E-05 | 0.02870027 | 0.143015 |
| chr2 | rs17736503 | G/T | intronic | *NCOA1* | Novel | Novel | 6.91E-05 | 0.03004851 | 0.117841 |
| chr14 | rs12895330 | G/C | intergenic | *AKAP6, NPAS3* | Novel | Novel, novel | 4.71E-05 | 0.03148983 | 0.146985 |
| chr10 | rs1247117 | G/A | intergenic | *PRLHR, CACUL1* | Novel | Novel, novel | 3.47E-06 | 0.03591847 | 0.161364 |
| chr1 | rs522367 | T/C | intronic | *SEC16B* | Novel | Confirmed | 4.36E-06 | 0.03615614 | 0.173609 |
| chr12 | rs35449 | T/G | intergenic | *TBX3, MED13L* | Novel | Novel | 5.55E-06 | 0.03941978 | 0.186513 |

Notes: The SNPs are listed with their chromosomal, allele, role, annotated genes, original *p*-value, $\mathrm{cFDR}$ and fdr.GPA values. A means CAD and B means CBMI. SNP type refers to whether the SNP identified in our study compared to the previous CBMI–related GWASs is novel or confirmed, or in high LD block with previously GWASs confirmed SNPs. Gene type refers to whether the gene identified in our study compared to the previous CBMI-related studies is novel or confirmed.

Chr: chromosome, cFDR: conditional false discovery rate, fdr.GPA: false discovery rate of GPA, GPA: genetic analysis incorporating pleiotropy and annotation, LD: linkage disequilibrium, CAD: coronary artery disease, CBMI: childhood body mass index.
